# Role of non-coding RNAs in non-aging-related neurological disorders

**DOI:** 10.1590/1414-431X20187566

**Published:** 2018-06-11

**Authors:** A.S. Vieira, D.B. Dogini, I. Lopes-Cendes

**Affiliations:** 1 Departamento de Biologia Estrutural e Funcional, Instituto de Biologia, Universidade Estadual de Campinas, Campinas, SP, Brasil; 2 Departamento de Genética Médica, Faculdade de Ciências Médicas, Universidade Estadual de Campinas, Campinas, SP, Brasil; 3 Instituto Brasileiro de Neurociência e Neurotecnologia, Campinas, SP, Brasil

**Keywords:** microRNA, Gene regulation, Molecular biomarkers

## Abstract

Protein coding sequences represent only 2% of the human genome. Recent advances have demonstrated that a significant portion of the genome is actively transcribed as non-coding RNA molecules. These non-coding RNAs are emerging as key players in the regulation of biological processes, and act as "fine-tuners" of gene expression. Neurological disorders are caused by a wide range of genetic mutations, epigenetic and environmental factors, and the exact pathophysiology of many of these conditions is still unknown. It is currently recognized that dysregulations in the expression of non-coding RNAs are present in many neurological disorders and may be relevant in the mechanisms leading to disease. In addition, circulating non-coding RNAs are emerging as potential biomarkers with great potential impact in clinical practice. In this review, we discuss mainly the role of microRNAs and long non-coding RNAs in several neurological disorders, such as epilepsy, Huntington disease, fragile X-associated ataxia, spinocerebellar ataxias, amyotrophic lateral sclerosis (ALS), and pain. In addition, we give information about the conditions where microRNAs have demonstrated to be potential biomarkers such as in epilepsy, pain, and ALS.

## Introduction

Recent developments have indicated that numerous non-coding sequences present in the human genome are actively transcribed as non-coding RNA (ncRNA) molecules (1). These ncRNAs may be grouped into different classes and classified according to size and function. They have emerged as key players in the regulation of many biological processes and the fine-tune control of gene expression (2).

It is not surprising that the complexity of neurological disorders is determined by different molecular mechanisms, including genetic mutations and epigenetic factors. In this context, changes in ncRNA gene expression regulation have emerged as a putative mechanism in a variety of neurological disorders such as epilepsy, neurodegenerative disorders, and autoimmune conditions (3,4). Specific processes by which ncRNAs may influence disease vary widely and include quantitative changes in coding and ncRNA expression, induction of abnormal RNA species, and others (2,5). Furthermore, circulating ncRNAs may act as disease biomarkers, contributing to early disease diagnosis and treatment follow-up (6).

In this review, we discuss the classification, biogenesis, and mechanisms of action of ncRNAs. We also review key studies that show associations between microRNA (miRNA) and long non-coding RNA (lncRNA) dysregulation and different early and adult onset neurological disorders, as well as the use of circulating miRNAs as biomarkers and potential therapeutic strategies based on manipulating ncRNAs. The role of ncRNAs in aging-related neurological disorders, such as Alzheimer's or Parkinson's disease, are thoroughly reviewed elsewhere and are not the focus of the present review (7–9).

## Structure, function, and classification of non-coding RNAs

ncRNAs are defined as RNA molecules transcribed from genomic DNA that are not translated into proteins (10). The earliest recognized members of this category of RNA molecules were transfer RNAs (tRNAs) and ribosomal RNAs (rRNAs) (10). More recently, an increasing number of other ncRNAs have been detected and characterized, leading to the discovery that at least two thirds of the mammalian genome is actively transcribed (1).

ncRNAs are, in a broader sense, classified as long or small RNAs. lncRNAs are molecules ranging from ∼200 nucleotides (nt) to more than 20 kilobases. The major components of this category are rRNAs, tRNAs, X-chromosome inactivation RNAs (XIST RNAs) and regulatory lncRNAs (2). However, lncRNAs are an ever-increasing category, with more components than the four mentioned above (2). Small ncRNAs have lengths ranging from 20 to 200 nt, including small regulatory miRNAs, small nucleolar RNAs (snoRNAs), and piwi interacting RNAs (piRNAs) (11,12).

**Figure 1.**
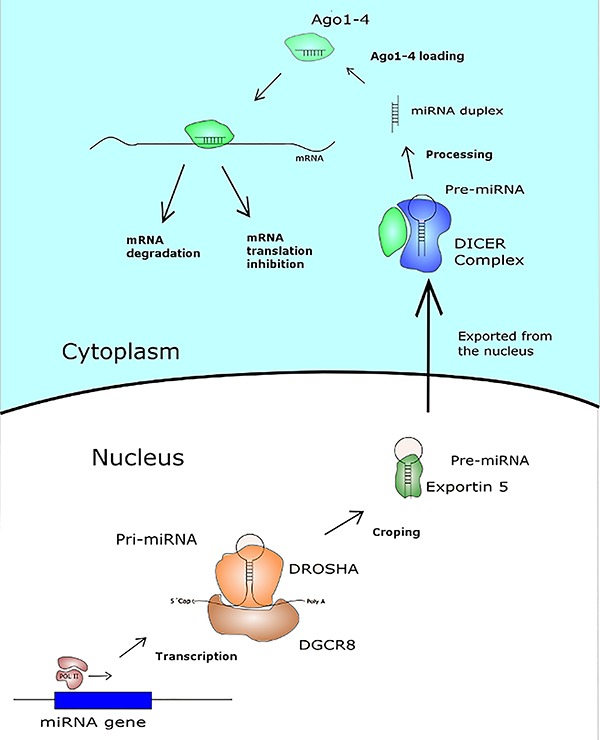
Main processes involved in the biogenesis and mechanism of action of microRNAs. DROSHA: Drosha ribonuclease III; DICER: Dicer 1; Ago1-4: Argonaute 1-4.

The molecular machinery responsible for miRNA biogenesis and interaction with mRNAs (Figure 1) is better elucidated than that underlying the activity of other ncRNAs. miRNA genes are transcribed by RNA polymerase II or III. This process generates a molecule, the pri-miR, that folds itself into a hairpin conformation and is 5′ capped and 3′ polyadenylated (13,14). The pri-miR molecule is recognized by the DROSHA RNAse III enzyme and cleaved, forming a 60- to 100-nt hairpin molecule, the pre-miR, that is exported from the nucleus to the cytoplasm (14,15). In the cytoplasm, the pre-miR is cleaved by the DICER enzyme, yielding a double-stranded ∼22nt RNA molecule (16). One of the strands of the formed 22-nt miRNA molecule is loaded into an RNA-induced silencing complex (RISC) protein to serve as the template for target mRNA recognition (17).

Mature miRNA molecules loaded into RISCs have two mechanisms of action. Perfect or near-perfect base pairing of the entire miRNA molecule to a complementary region within an mRNA leads to mRNA degradation by RISC (18). Perfect base pairing of almost all 22 nt is an uncommon scenario in animals. The more common scenario involves imperfect pairing, or pairing of a 5–8 nt ‘seed’ region of the miRNA, which leads to reduced translation or destabilization of the target mRNA (19). A single miRNA molecule may regulate multiple genes that contain a sequence complementary to the miRNA seed, and a given mRNA may be regulated by different miRNAs (20). Notably, the administration of exogenous nucleic acid sequences can mimic miRNA action (mimic-miRs), and employ the endogenous cellular machinery for miRNA-mediated gene silencing (21). Another possibility is the administration of stabilized exogenous nucleic acid sequences that are complementary to endogenous miRNAs, such as antagomirs, resulting in the inhibition of target cellular miRNAs (22).

miRNAs are also present and enriched in the plasma and serum. Furthermore, these RNAs are especially resistant to degradation (23). Blood circulating miRNAs are contained in microvesicles known as exosomes or are associated with Argonaute 2 complexes and, as a consequence, are protected from degradation (6,24). Because circulating miRNAs may originate from many different tissues throughout the body and may reflect normal function, changes in the circulating levels of these miRNAs may constitute a useful and easily accessible biomarker of many different pathological conditions. Moreover, it is feasible to quantify the levels of such circulating miRNAs by RT-PCR or even high throughput techniques such as micro-arrays or RNA-sequencing. The dysregulation of miRNA expression is well established in some tumors, and circulating miRNAs are indeed emerging as promising biomarkers in this field (23,25). The search for circulating miRNAs as biomarkers is also being applied to neurological disorders.

**Figure 2.**
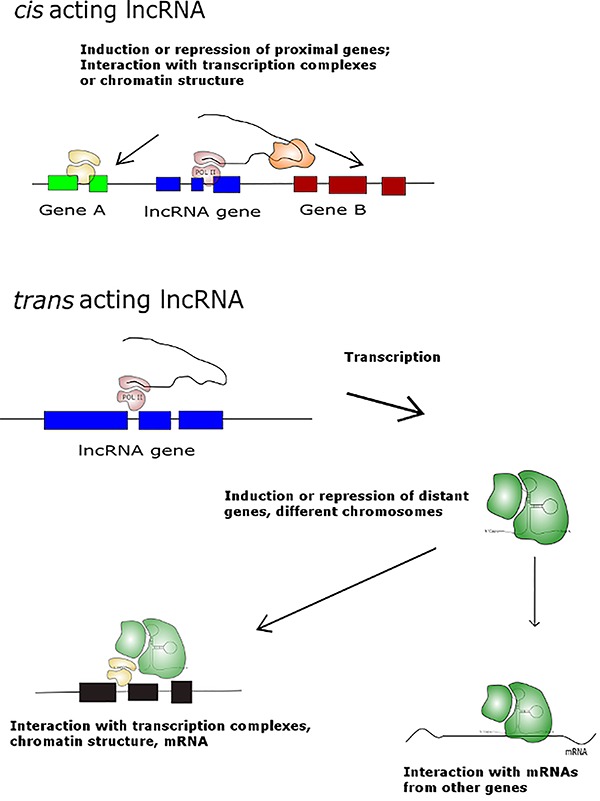
Mechanisms by which long non-coding RNAs (lncRNAs) can regulate gene expression.

lncRNAs boast distinct and diverse molecular machinery involved in the regulation of gene expression (Figure 2). Most of these ncRNAs are RNA polymerase II products that lack open reading frames but are generally 5′ capped and 3′ polyadenylated (26,27). lncRNAs are numerous, with estimates in the range of thousands of lncRNA coding genes (28). Briefly, lncRNAs may act in *cis*, silencing or enhancing the expression of proximal genes on the same chromosome. For example, the lncRNA HOTTIP gene is present in the HOXA gene cluster, and its expression enhances the expression of other component genes in the same cluster (20). lncRNAs may also act in *trans*, silencing or enhancing the expression of genes on different chromosomes. One example of an lncRNA acting in *trans* is Six3OS. This lncRNA was shown to activate the targets of the retinal development involving the Six3 transcription factor (29). Another mechanism of action for lncRNAs is the regulation of other ncRNAs. lncRNA can act as a ‘sponge' or decoy target. The lncRNA lincRNA-RoR mechanism of action illustrates this mechanism: this lncRNA has a binding site for miR-145, and the presence of lincRNA-RoR inhibits miR-145 action by interacting directly with lncRNA miRNA (30). The mechanisms of lncRNA-mediated regulation of protein-coding gene transcription are explored in more detail in the current literature (26,27).

## Role of non-coding RNAs in disease

Table 1 presents a list of ncRNAs associated with mechanisms underlying selected neurological disorders.

**Table 1.** List of ncRNAs associated with different mechanisms underlying selected neurological disorders.

Disorder	Gene Affected	Proposed mechanisms associated with Noncoding RNAs	References
FXTAS	*FMR1; FMR4*	Sequestration of RNA binding protein; antisense transcript	Tassone et al. 2004 58
DM1	*DMPK*	Sequestration of RNA binding protein; antisense transcript	Rau et al. 2011 66
SCA1	*ATXN1*	Altered miRNA pathway	Galka-Marciniak et al. 2012 56
SCA3	*ATXN3*	An auxiliary toxic long CAG repeat RNA; altered miRNA pathway	Galka-Marciniak et al. 2012 56
SCA7	*ATXN7*	Antisense transcript repress sense ataxin-7	Tan et al. 2014 63
SCA8	*ATXN8OS; ATXN8*	Sequestration of RNA binding protein; antisense transcript	Daughters et al. 2009 61; Moseley et al. 2006 62
HDL2	*JPH3*	Antisense transcript; polyQ toxicity	Wojciechowska and Krzyzosiak, 2011 5
MTLE	*P2X7*	Down-regulation by miR-22	Jimenez-Mateos et al. 2015 3
HD	*HTT*	An auxiliary toxic long CAG repeat RNA; altered miRNA pathway	Wojciechowska and Krzyzosiak, 2011 5
MTLE	Genes involved with inflammation	Up-regulation of miR-146a expression	Aronica et al. 2010 37
ALS	*SOD1* and others	An artificial microRNA may extend survival and delays paralysis; Up regulation of miR-206.	Stoica et al. 2016 79; Takahashi et al. 2015 81
Cortical dysplasia	*Lis1*	Dysregulation of miR-139-5p	Huang et al. 2014 90
Pain	Inflammation, neural processing	Dysregulation of miR-1, -16, and -206	Kusuda et al. 2011 86

### Epilepsy

Epilepsy is a neurological condition with a high prevalence in the population (1.5–2%). A common feature of different epileptic conditions is the occurrence of seizures (31,32). The mechanism responsible for epileptogenesis (the process by which normal nervous tissue becomes epileptic) is complex and multifactorial (33). Evidence in the literature, as reviewed below, indicates that ncRNAs may have critical roles in the molecular mechanisms associated with epilepsy (34).

Hippocampal tissue from patients with mesial temporal lobe epilepsy (MTLE) who underwent temporal lobe resection for the control of seizures has been shown to have a reduction in the overall expression of miRNAs when compared with normal hippocampus from autopsy controls (35). Moreover, MTLE is associated with inflammation, and changes in the expression of miRNAs involved in the regulation of inflammation have been demonstrated in samples from MTLE patients (36,37). For example, miR-146-a, a miRNA involved in inflammation, is upregulated in resected hippocampus from MTLE patients (37).

In animal models of epilepsy, the dysregulation of miRNAs has been explored more extensively. miRNA expression studies were performed, using high-throughput platforms, in the animal model induced by lithium-pilocarpine, systemic kainic acid, and by intra-amygdalar kainic acid injection (38–40). Based on such studies, an extensive list of candidate miRNAs was found, but relatively few miRNAs were consistent among different studies. One example of replicable findings is mir-34a, which was found to be differentially expressed in two independent studies (38,41). mir-134 is another promising miRNA that may be involved in the molecular mechanisms of epilepsy. mir-134 was found to be differentially expressed in an epilepsy animal model, and the reduction in its expression by antagomir administration was shown to reduce cell death and seizure severity (42). In addition, downregulation of mir-132 in an animal model reduced seizure-induced neuronal death (40).

More recently, Jimenez-Mateos et al. (3) demonstrated that miR-22 downregulates the purinergic P2X7 receptor, a key component of the inflammatory response, in a mouse model of focal onset status-epilepticus. Furthermore, an increase in miR-22 activity by the administration of a Mir-22 mimic molecule reduced spontaneous seizures in these mice (3).

The role of lncRNAs has also been explored in the context of experimental animal models of epilepsy. Lee et al. (43) explored the expression of lncRNAs in two animal epilepsy models, pilocarpine- and kainic acid-induced seizures (43). These authors found hundreds of lnRNAs that were differentially expressed when comparing nervous tissue from controls with that of treated mice. Of these differentially expressed lncRNAs, 54 (for pilocarpine) and 14 (for kainic acid) were close to protein-coding genes and appear to induce significant changes in gene expression, thus indicating a possible *cis* effect of these lncRNAs (43).

The first evidence for the potential use of miRNAs as biomarkers in epilepsy also came from studies in experimental animal models. Liu et al. (44) demonstrated the differential regulation of several miRNAs isolated from the blood of rats that received the chemoconvulsant kainic acid. More recently, Roncon et al. (45) found 27 miRNAs to be differentially expressed in the plasma of rats treated with pilocarpine. In humans, Wang et al. (46), using RNA-sequencing and subsequent RT-PCR validation, found four upregulated and two downregulated blood circulating miRNAs when comparing epilepsy patients to healthy controls. Among the differentially expressed miRNAs, miR-106b-5p had the highest sensitivity and specificity (46). Furthermore, in a subsequent study, there were five circulating miRNAs identified as potential biomarkers of drug-resistant epilepsy, and miR-301a-3p had the highest sensitivity and specificity (47). We have identified that miR-134 is a circulating biomarker for patients with mesial temporal lobe epilepsy regardless of their response to treatment, which may help in the diagnosis of this type of epilepsy (48).

In focal cortical dysplasia, a cortical malformation frequently associated with refractory seizures, miR-4521 has been shown to be upregulated in the plasma of patients compared to control subjects (49).

### Neurodegenerative and neuromuscular disorders

Neurodegenerative disorders are associated with a wide range of genetic mutations and epigenetic and environmental factors. Among genetic mutations, trinucleotide repeat expansion is increasingly recognized as the cause of a large subset of these conditions. Trinucleotide repeat expansions account for more than 30 neurological and neuromuscular diseases that are categorized into coding and non-coding repeat expansion disorders, depending on the genetic location of their causative mutations (50–52). Disorders such as Huntington's disease (HD), spinocerebellar ataxia (SCA) types 1, 2, 3, 6, 7, 8, and 17, dentatorubral-pallidoluysian atrophy, and spinal and bulbar muscular atrophy are typically associated with a protein gain-of-function mechanism (53). In contrast, diseases such as myotonic dystrophy type 1 (DM1) (54,55), fragile X-associated tremor ataxia syndrome (FXTAS), myotonic dystrophy type 2 (DM2), SCA31, SCA10, SCA8, and, more recently, amyotrophic lateral sclerosis and frontotemporal sclerosis have been associated with an RNA gain-of-function mechanism in which the trinucleotide expansion leads to the formation of nuclear RNA foci that sequester specific RNA-binding proteins (5,56,57).

**Figure 3.**
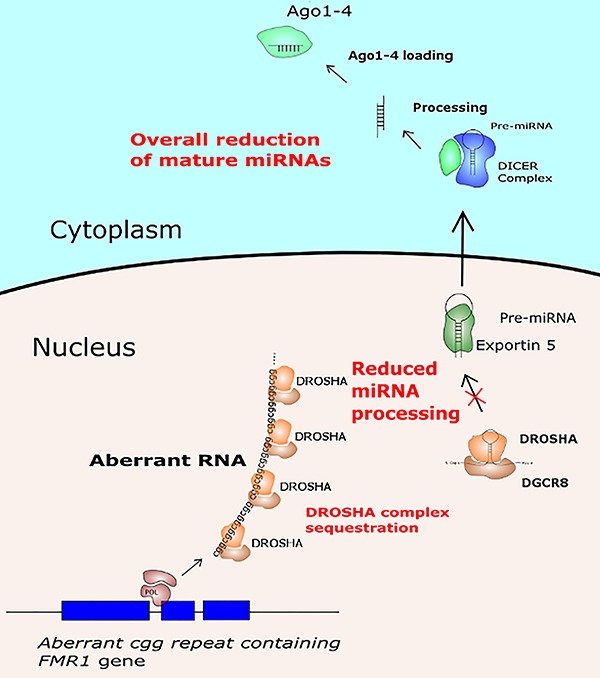
Mechanism involved in microRNA machinery sequestration by aberrant RNA species produced in a triplet repeat disease, fragile-X associated tremor ataxia syndrome (FXTAS). DICER: Dicer 1; DROSHA: Drosha ribonuclease III; Ago1-4: Argonaute 1-4.

Studies of FXTAS have established that the sequestration of RNA-binding proteins due to the expression of pathogenic RNA with expanded repeats is involved in disease pathogenesis (58) (Figure 3). A recent study identified that the double-stranded RNA-binding protein DGCR8 binds to expanded CGG repeats, resulting in the partial sequestration of DGCR8 and its partner, DROSHA, within CGG RNA aggregates. Consequently, the processing of miRNAs is reduced, resulting in decreased levels of mature miRNAs in neuronal cells expressing expanded CGG repeats such as in brain tissue from patients with FXTAS (59).

SCA8 is a dominantly inherited, slowly progressive neurodegenerative disorder caused by a CTG CAG repeat expansion (60). In pathological samples from SCA8 patients, bidirectional (sense and antisense) expression of the SCA8 CTG·CAG expansion produces toxic non-coding CUG expansion in RNAs from the Ataxin 8 opposite strand (ATXN8OS) and a nearly pure polyglutamine expansion protein encoded by ATXN8 (61,62). In SCA7, the tissue-specific alterations caused by CAG repeat expression in the ATXN7 gene seems to be related to cross-talk between the lncRNA lnc-SCA7, the ATXN7 mRNA, and mir-124. Mutant ATXN7 disrupts this crosstalk and is itself upregulated, since it is not repressed by ncRNAs (63).

Recent studies have suggested that alterations in small regulatory ncRNAs, such as miRNAs, could contribute to the pathogenesis of several neurodevelopmental disorders. Some studies have found a relationship between miRNAs and DM1 (64). Alterations in the miRNA expression patterns have been observed in muscle-specific miRNAs (myomiRs). Given the small distance between the seed binding sites of miR-206 and 148a in the DMPK 3′ UTR, Koscianska et al. (65) analyzed the binding mechanism of both miRNAs. They discovered cooperative binding; the joint binding of miRs 206 and 148a increased the negative regulation of DMPK mRNA. These findings provide mechanistic insights into the miRNA-mediated regulation of the DMPK transcript. In this regard, the dysregulation of DM1-associated miRNAs has also been linked to alterations in their predictive target expression, showing that miRNA dysregulation in DM1 is functionally relevant and may contribute to disease pathology (66,67). Furthermore, RNA toxicity has been confirmed in transgenic mice harboring long triplet repeats in the *dmpk* gene. Seznec et al. (68) showed that mice develop multi-system abnormalities mimicking the human DM phenotype, with predominant involvement of muscles and the central nervous system (CNS). Pathway and function analysis highlighted the involvement of the miRNA-dysregulated mRNAs in multiple aspects of DM2 pathophysiology as well (4,69).

Huntington's disease is characterized by widespread mRNA dysregulation, especially in the striatum and cortical regions and alterations in miRNA-mediated post-transcriptional regulation could be an important mechanism contributing to mRNA dysregulation in HD (70). In addition, there is evidence that abnormal neurodevelopment might also have a critical role in HD (71). These emerged from studies using mouse embryonic stem cells and patient-derived induced pluripotent stem cells (The HD iPSC Consortium, 2012) showing that chromatin modifications and DNA methylation status support the hypothesis that wild-type and mutant Huntingtin might affect key chromatin regulators such as DNA and histone methyltransferases, and demethylases (72–74). In fact, a growing body of evidence suggests that alterations of epigenetic modifications constitute a basic molecular mechanism caused by the HD mutation and are responsible for early features of the pathological process (75). Furthermore, a recent genome-wide screen of miRNAs in *post mortem* brains highlighted miRNAs that were differentially expressed in HD patients, especially miRNAs in the HOX family, which have been associated with early brain development (76). Indeed, there are several classes of lncRNAs that are potentially involved in developmental processes and that were found to be dysregulated in brain tissue from patients with HD such as TUG1, NEAT1, MEG3, and DGCR5 (77).

Amyotrophic lateral sclerosis (ALS) is a widespread motor neuron disorder causing injury and death of lower and upper motor neurons. Familial ALS (∼10% of all ALS cases) is inherited as a dominant trait, and 20% of these cases have mutations in the gene encoding Cu/Zn cytosolic superoxide dismutase 1 (*SOD1*) (78). A recent study demonstrated that an AAV9-delivered SOD1-specific artificial miRNA is an effective and translatable therapeutic approach to ALS (79). Another promising miRNA with a possible therapeutic use in ALS is mir-155. It was demonstrated that this inflammation-associated miRNA is upregulated in the mutant SOD1 mouse model and that reduction in the expression of mir-155 significantly extended the life span of this mouse (80).

In addition, expression levels of certain miRNAs, such as miR-4649-5p and hsa-miR-4299, were significantly correlated with disease progression and might be useful as prognostic biomarkers (81). Another potential biomarker was mir-206, found to be upregulated in the plasma of SOD1-G93A mice, an experimental ALS model, and in patients with confirmed ALS (82). In addition, there is evidence of dysregulation of miRNAs extracted from leukocytes from sporadic ALS patients (83). More recently, we have demonstrated that among 11 miRNAs identified as differently expressed in muscle of patients with ALS, only two, miR-214 and miR-424, correlated with clinical deterioration over time in these patients (84).

### Pain

Conditions leading to chronic pain are related to multiple etiologic factors, ranging from maladaptive neuronal plasticity to diverse inflammatory pathways (85). Due to the complexity of chronic pain, some studies have explored the possible role of ncRNAs in different experimental pain models. Kusuda et al. (86) observed a change in the expression of three miRNAs, miRs 1, 16, and 206, in different pain conditions such as peripheral inflammation, nerve ligation, or axotomy. Other studies have employed low-density TaqMan arrays to profile the expression pattern of miRNAs after spinal nerve ligation in rats and found 63 altered miRNAs (87).

A possible role for lncRNAs has been explored in experimental models of neuropathic pain. A microarray analysis demonstrated hundreds of differentially expressed lncRNAs and mRNAs in the spinal cords of mice subjected to spinal nerve ligation. As demonstrated in other experiments, 35 differentially regulated lncRNAs were in genomic regions proximal to differentially regulated genes from the same dataset (88).

## Non-coding RNAs as target treatments for neurologic disorders

The use of ncRNAs as therapeutic tools in human disorders is still in its early stages. To date, there is only one therapeutic use of human miRNA for the treatment of hepatitis C (HCV) that has passed phase IIa clinical trials (89). The clinical trial data showed the efficacy of the employed anti-miRNA in reducing viral load and showed good treatment tolerability, thus indicating the feasibility of similar strategies for other clinical uses such as in the case of neurological conditions.

Animal experiments already indicate some promising targets for the use of ncRNAs as therapeutic tools in disorders affecting the CNS. In epilepsy, the use of miR antagonists for miR-134 or mimic-miRs for miR-22 was capable of reducing neuronal death and seizure severity in animal models (3,42). These and other examples of pre-clinical uses of miRNAs for the treatment of neurological conditions need further study; however, due to the good tolerability already shown in the existing human clinical trial for HCV, there is optimism about the possible utility of ncRNAs in the treatment of neurological conditions in the future. However, several challenges remain for the efficient delivery of ncRNA molecules into the CNS, thus most of the pre-clinical studies still use invasive techniques for administering these molecules (4,44)

## Conclusions

In conclusion, ncRNAs are emerging as key players in the field of neurological disorders. ncRNAs are involved in many conditions, either as part of the molecular mechanisms underlying disease or as biomarkers that may be used for improved diagnosis or assessment of disease progression. ncRNAs are also promising targets for new therapeutic strategies to be employed in the treatment of neurological conditions.

## References

[1] Djebali S, Davis CA, Merkel A, Dobin A, Lassmann T, Mortazavi A (2012). Landscape of transcription in human cells. Nature.

[2] Peschansky VJ, Wahlestedt C (2014). Non-coding RNAs as direct and indirect modulators of epigenetic
regulation. Epigenetics.

[3] Jimenez-Mateos EM, Arribas-Blazquez M, Sanz-Rodriguez A, Concannon C, Olivos-Ore LA, Reschke CR (2015). microRNA targeting of the P2X7 purinoceptor opposes a
contralateral epileptogenic focus in the hippocampus. Sci Rep.

[4] Greco S, Perfetti A, Fasanaro P, Cardani R, Capogrossi MC, Meola G (2012). Deregulated microRNAs in myotonic dystrophy type
2. PloS One.

[5] Wojciechowska M, Krzyzosiak WJ (2011). Cellular toxicity of expanded RNA repeats: focus on RNA
foci. Hum Mol Genet.

[6] Arroyo JD, Chevillet JR, Kroh EM, Ruf IK, Pritchard CC, Gibson DF (2011). Argonaute2 complexes carry a population of circulating microRNAs
independent of vesicles in human plasma. Proc Natl Acad Sci USA.

[7] Femminella GD, Ferrara N, Rengo G (2015). The emerging role of microRNAs in Alzheimer's
disease. Front Physiol.

[B08] Zhang Z (2016). Long non-coding RNAs in Alzheimer's disease. Curr Top Med Chemy.

[9] Majidinia M, Mihanfar A, Rahbarghazi R, Nourazarian A, Bagca B, Avci CB (2016). The roles of non-coding RNAs in Parkinson's
disease. Mol biol rep.

[10] Huttenhofer A, Schattner P, Polacek N (2005). Non-coding RNAs: hope or hype?. Trends Genet.

[11] Mattick JS, Makunin IV (2006). Non-coding RNA. Hum Mol Genet.

[12] Megosh HB, Cox DN, Campbell C, Lin H (2006). The role of PIWI and the miRNA machinery in Drosophila germline
determination. Curr Biol.

[13] McNeill E, Van Vactor D (2012). MicroRNAs shape the neuronal landscape. Neuron.

[14] Kim VN (2005). MicroRNA biogenesis: coordinated cropping and
dicing. Nat Rev Mol Cell Biol.

[15] Lee Y, Ahn C, Han J, Choi H, Kim J, Yim J (2003). The nuclear RNase III Drosha initiates microRNA
processing. Nature.

[16] Hutvágner G, McLachlan J, Pasquinelli AE, Bálint E, Tuschl T, Zamore PD (2001). A cellular function for the RNA-interference enzyme Dicer in the
maturation of the let-7 small temporal RNA. Science.

[17] Du T, Zamore PD (2005). microPrimer: the biogenesis and function of
microRNA. Development.

[18] Gu S, Kay MA How do miRNAs mediate translational repression?. Silence.

[19] Cannell IG, Kong YW, Bushel M (2008). How do microRNAs regulate gene expression?. Biochem Soc Trans.

[20] Wang KC, Yang YW, Liu B, Sanyal A, Corces-Zimmerman R, Chen Y (2011). A long noncoding RNA maintains active chromatin to coordinate
homeotic gene expression. Nature.

[21] Wang Z (2011). The guideline of the design and validation of MiRNA
mimics. Methods Mol Biol.

[22] Krutzfeldt J, Rajewsky N, Braich R, Rajeev KG, Tuschl T, Manoharan M (2005). Silencing of microRNAs in vivo with ‘antagomirs'. Nature.

[23] Chen X, Ba Y, Ma L, Cai X, Yin Y, Wang K (2008). Characterization of microRNAs in serum: a novel class of
biomarkers for diagnosis of cancer and other diseases. Cell Res.

[24] Valadi H, Ekstrom K, Bossios A, Sjostrand M, Lee JJ, Lotvall JO (2007). Exosome-mediated transfer of mRNAs and microRNAs is a novel
mechanism of genetic exchange between cells. Nat Cell Biol.

[25] Toiyama Y, Takahashi M, Hur K, Nagasaka T, Tanaka K, Inoue Y (2013). Serum miR-21 as a diagnostic and prognostic biomarker in
colorectal cancer. J Nat Cancer Inst.

[26] Kornienko AE, Guenzl PM, Barlow DP, Pauler FM (2013). Gene regulation by the act of long non-coding RNA
transcription. BMC Biol.

[27] Ulitsky I, Bartel DP (2013). lincRNAs: genomics, evolution, and mechanisms. Cell.

[28] Derrien T, Johnson R, Bussotti G, Tanzer A, Djebali S, Tilgner H (2012). The GENCODE v7 catalog of human long noncoding RNAs: analysis of
their gene structure, evolution, and expression. Genome Res.

[29] Rapicavoli NA, Poth EM, Zhu H, Blackshaw S (2011). The long noncoding RNA Six3OS acts in trans to regulate retinal
development by modulating Six3 activity. Neural Dev.

[30] Wang Y, Xu Z, Jiang J, Xu C, Kang J, Xiao L (2013). Endogenous miRNA sponge lincRNA-RoR regulates Oct4, Nanog, and
Sox2 in human embryonic stem cell self-renewal. Dev Cell.

[31] Engel J (2001). Mesial temporal lobe epilepsy: what have we
learned?. The Neuroscientist.

[32] Annegers JF, Rocca WA, Hauser WA (1996). Causes of epilepsy: contributions of the Rochester epidemiology
project. Mayo Clin Proc.

[33] Pitkänen A, Lukasiuk K (2011). Mechanisms of epileptogenesis and potential treatment
targets. Lancet Neurol.

[34] Dogini DB, Avansini SH, Vieira AS, Lopes-Cendes I (2013). MicroRNA regulation and dysregulation in epilepsy. Front Cell Neurosci.

[35] McKiernan RC, Jimenez-Mateos EM, Bray I, Engel T, Brennan GP, Sano T (2012). Reduced mature microRNA levels in association with dicer loss in
human temporal lobe epilepsy with hippocampal sclerosis. PloS One.

[36] Vezzani A, Friedman A, Dingledine RJ (2013). The role of inflammation in epileptogenesis. Neuropharmacology.

[37] Aronica E, Fluiter K, Iyer A, Zurolo E, Vreijling J, van Vliet EA (2010). Expression pattern of miR-146a, an inflammation-associated
microRNA, in experimental and human temporal lobe epilepsy. Euro J Neurosci.

[38] Hu K, Xie YY, Zhang C, Ouyang DS, Long HY, Sun DN (2012). MicroRNA expression profile of the hippocampus in a rat model of
temporal lobe epilepsy and miR-34a-targeted neuroprotection against
hippocampal neurone cell apoptosis post-status epilepticus. BMC Neurosci.

[B39] McKiernan RC, Jimenez-Mateos EM, Sano T, Bray I, Stallings RL, Simon RP (2012). Expression profiling the microRNA response to epileptic
preconditioning identifies miR-184 as a modulator of seizure-induced
neuronal death. Exp Neurol.

[40] Jimenez-Mateos EM, Bray I, Sanz-Rodriguez A, Engel T, McKiernan RC, Mouri G (2011). miRNA Expression profile after status epilepticus and hippocampal
neuroprotection by targeting miR-132. Am J Pathol.

[41] Sano T, Reynolds JP, Jimenez-Mateos EM, Matsushima S, Taki W, Henshall DC (2012). MicroRNA-34a upregulation during seizure-induced neuronal
death. Cell Death Dis.

[42] Jimenez-Mateos EM, Engel T, Merino-Serrais P, McKiernan RC, Tanaka K, Mouri G (2012). Silencing microRNA-134 produces neuroprotective and prolonged
seizure-suppressive effects. Nat Med.

[43] Lee DY, Moon J, Lee ST, Jung KH, Park DK, Yoo JS (2015). Dysregulation of long non-coding RNAs in mouse models of
localization-related epilepsy. Biochem Biophys Res Commun.

[44] Liu DZ, Tian Y, Ander BP, Xu H, Stamova BS, Zhan X (2010). Brain and blood microRNA expression profiling of ischemic stroke,
intracerebral hemorrhage, and kainate seizures. J Cereb Blood Flow Metab.

[45] Roncon P, Soukupova M, Binaschi A, Falcicchia C, Zucchini S, Ferracin M (2015). MicroRNA profiles in hippocampal granule cells and plasma of rats
with pilocarpine-induced epilepsy--comparison with human epileptic
samples. Sci Rep.

[46] Wang J, Yu JT, Tan L, Tian Y, Ma J, Tan CC (2015). Genome-wide circulating microRNA expression profiling indicates
biomarkers for epilepsy. Sci Rep.

[47] Wang J, Tan L, Tan L, Tian Y, Ma J, Tan CC (2015). Circulating microRNAs are promising novel biomarkers for
drug-resistant epilepsy. Sci Rep.

[48] Avansini SH, de Sousa Lima BP, Secolin R, Santos ML, Coan AC, Vieira AS (2017). MicroRNA hsa-miR-134 is a circulating biomarker for mesial
temporal lobe epilepsy. PLoS One.

[49] Wang X, Sun Y, Tan Z, Che N, Ji A, Luo X (2016). Serum MicroRNA-4521 is a Potential Biomarker for Focal Cortical
Dysplasia with Refractory Epilepsy. Neurochem Res.

[50] Lopez Castel A, Cleary JD, Pearson CE (2010). Repeat instability as the basis for human diseases and as a
potential target for therapy. Nat Rev Mol Cell Biol.

[B51] Mirkin SM (2007). Expandable DNA repeats and human disease. Nature.

[52] Ranum LP, Day JW (2002). Dominantly inherited, non-coding microsatellite expansion
disorders. Curr Opin Genet Dev.

[53] Orr HT, Zoghbi HY (2007). Trinucleotide repeat disorders. Ann Rev Neurosci.

[54] Wang LC, Chen KY, Pan H, Wu CC, Chen PH, Liao YT (2011). Muscleblind participates in RNA toxicity of expanded CAG and CUG
repeats in Caenorhabditis elegans. Cell Mol Life Sci.

[55] Mykowska A, Sobczak K, Wojciechowska M, Kozlowski P, Krzyzosiak WJ (2011). CAG repeats mimic CUG repeats in the misregulation of alternative
splicing. Nucleic Acids Res.

[56] Galka-Marciniak P, Urbanek MO, Krzyzosiak WJ (2012). Triplet repeats in transcripts: structural insights into RNA
toxicity. Biol Chem.

[57] Gendron TF, Belzil VV, Zhang YJ, Petrucelli L (2014). Mechanisms of toxicity in C9FTLD/ALS. Acta Neuropathol.

[58] Tassone F, Iwahashi C, Hagerman PJ (2004). FMR1 RNA within the intranuclear inclusions of fragile
X-associated tremor/ataxia syndrome (FXTAS). RNA Biol.

[59] Sellier C, Freyermuth F, Tabet R, Tran T, He F, Ruffenach F (2013). Sequestration of DROSHA and DGCR8 by expanded CGG RNA repeats
alters microRNA processing in fragile X-associated tremor/ataxia
syndrome. Cell Rep.

[60] Ikeda Y, Shizuka-Ikeda M, Watanabe M, Schmitt M, Okamoto K, Shoji M (2000). Asymptomatic CTG expansion at the SCA8 locus is associated with
cerebellar atrophy on MRI. J Neurol Sci.

[61] Daughters RS, Tuttle DL, Gao W, Ikeda Y, Moseley ML, Ebner TJ (2009). RNA gain-of-function in spinocerebellar ataxia type
8. PLoS Genet.

[62] Moseley ML, Zu T, Ikeda Y, Gao W, Mosemiller AK, Daughters RS (2006). Bidirectional expression of CUG and CAG expansion transcripts and
intranuclear polyglutamine inclusions in spinocerebellar ataxia type
8. Nat Genet.

[63] Tan JY, Vance KW, Varela MA, Sirey T, Watson LM, Curtis HJ (2014). Cross-talking noncoding RNAs contribute to cell-specific
neurodegeneration in SCA7. Nat Struct Mol Biol.

[64] Turner C, Hilton-Jones D (2010). The myotonic dystrophies: diagnosis and
management. J Neurol Neurosurg Psychiatry.

[65] Koscianska E, Witkos TM, Kozlowska E, Wojciechowska M, Krzyzosiak WJ (2015). Cooperation meets competition in microRNA-mediated DMPK
transcript regulation. Nucleic Acids Res.

[66] Rau F, Freyermuth F, Fugier C, Villemin JP, Fischer M. C, Jost B (2011). Misregulation of miR-1 processing is associated with heart
defects in myotonic dystrophy. Nature Struct Molec Biol.

[67] Perbellini R, Greco S, Sarra-Ferraris G, Cardani R, Capogrossi MC, Meola G (2011). Dysregulation and cellular mislocalization of specific miRNAs in
myotonic dystrophy type 1. Neuromuscul Disorder.

[68] Seznec H, Agbulut O, Sergeant N, Savouret C, Ghestem A, Tabti N (2001). Mice transgenic for the human myotonic dystrophy region with
expanded CTG repeats display muscular and brain
abnormalities. Hum Mol Genet.

[69] Deng JH, Deng P, Lin SL, Ying SY (12015). Gene silencing in vitro and in vivo using intronic
microRNAs. Meth Molecular Biol.

[70] Hoss AG, Lagomarsino VN, Frank S, Hadzi TC, Myers RH, Latourelle JC (2015). Study of plasma-derived miRNAs mimic differences in Huntington's
disease brain. Mov Disorder.

[71] Humbert S (2010). Is Huntington disease a developmental disorder?. EMBO Rep.

[72] HD iPSC Consortium (2012). Induced pluripotent stem cells from patients with Huntington's
disease show CAG-repeat-expansion-associated phenotypes. Cell Stem Cell.

[B73] Ng CW, Yildirim F, Yap YS, Dalin S, Matthews BJ, Velez PJ (2013). Extensive changes in DNA methylation are associated with
expression of mutant huntingtin. Proc Natl Acad Sci USA.

[74] Biagioli M, Ferrari F, Mendenhall EM, Zhang Y, Erdin S, Vijayvargia R (2015). Htt CAG repeat expansion confers pleiotropic gains of mutant
huntingtin function in chromatin regulation. Hum Mol Genet.

[75] Kerschbamer E, Biagioli M (2015). Huntington's disease as neurodevelopmental disorder: altered
chromatin regulation, coding, and non-coding RNA
transcription. Front Neurosci.

[76] Hoss AG, Kartha VK, Dong X, Latourelle JC, Dumitriu A, Hadzi TC (2014). MicroRNAs located in the Hox gene clusters are implicated in
huntington's disease pathogenesis. PLoS Genet.

[77] Johnson R (2012). Long non-coding RNAs in Huntington's disease
neurodegeneration. Neurobiol Dis.

[78] Rosen DR, Siddique T, Patterson D, Figlewicz DA, Sapp P, Hentati A (1993). Mutations in Cu/Zn superoxide dismutase gene are associated with
familial amyotrophic lateral sclerosis. Nature.

[79] Stoica L, Todeasa SH, Toro Cabrera G, Salameh JS, ElMallah MK, Mueller C (2016). Adno associated virus delivered artificial microRNA extends
survival and delays paralysis in an amyotrophic lateral sclerosis mouse
model. Ann Neurol.

[80] Butovsky O, Jedrychowski MP, Cialic R, Krasemann S, Murugaiyan G, Fanek (2015). Targeting miR-155 restores abnormal microglia and attenuates
disease in SOD1 mice. Ann Neurol.

[81] Takahashi I, Hama Y, Matsushima M, Hirotani M, Kano T, Hohzen H (2015). Identification of plasma microRNAs as a biomarker of sporadic
amyotrophic lateral sclerosis. Mol Brain.

[82] Toivonen JM, Manzano R, Olivan S, Zaragoza P, Garcia-Redondo A, Osta R (2014). MicroRNA-206: a potential circulating biomarker candidate for
amyotrophic lateral sclerosis. PloS One.

[83] De Felice B, Guida M, Guida M, Coppola C, De Mieri G, Cotrufo R (2012). A miRNA signature in leukocytes from sporadic amyotrophic lateral
sclerosis. Gene.

[84] de Andrade HM, de Albuquerque M, Avansini SH, de S Rocha C, Dogini DB, Nucci A (2016). MicroRNAs-424 and 206 are potential prognostic markers in spinal
onset amyotrophic lateral sclerosis. J Neurol Sci.

[85] Basbaum AI, Bautista DM, Scherrer G, Julius D (2009). Cellular and molecular mechanisms of pain. Cell.

[86] Kusuda R, Cadetti F, Ravanelli MI, Sousa TA, Zanon S, De Lucca FL (2011). Differential expression of microRNAs in mouse pain
models. Mol Pain.

[87] von Schack D, Agostino MJ, Murray BS, Li Y, Reddy PS, Chen J (2011). Dynamic changes in the microRNA expression profile reveal
multiple regulatory mechanisms in the spinal nerve ligation model of
neuropathic pain. PloS One.

[88] Jiang BC, Sun WX, He LN, Cao DL, Zhang ZJ, Gao YJ (2015). Identification of lncRNA expression profile in the spinal cord of
mice following spinal nerve ligation-induced neuropathic
pain. Mol Pain.

[89] van der Ree MH, van der Meer AJ, de Bruijne J, Maan R, van Vliet A, Welzel TM (2014). Long-term safety and efficacy of microRNA-targeted therapy in
chronic hepatitis C patients. Antiviral Res.

[90] Huang Y, Jiang J, Zheng G, Chen J, Lu H, Guo H (2014). miR-139-5p modulates cortical neuronal migration by targeting
Lis1 in a rat model of focal cortical dysplasia. Int J Mol Med.

